# Neural network-based image analysis of co-localized microorganisms and human cells on implant materials

**DOI:** 10.1038/s41598-025-05484-1

**Published:** 2025-06-20

**Authors:** Nicolas Debener, Anna Rosner, Jannik Menke, Carina Mikolai, Meike Stiesch, Katharina Doll-Nikutta, Janina Bahnemann

**Affiliations:** 1 Institute of Technical Chemistry, Leibniz University Hannover, Hannover, Germany; 2 Department of Prosthetic Dentistry and Biomedical Materials Science, Hannover Medical School, Hannover, Germany; 3 Lower Saxony Center for Biomedical Engineering, Implant Research and Development (NIFE), Hannover, Germany; 4 Institute of Physics, University of Augsburg, Augsburg, Germany; 5 Centre for Advanced Analytics and Predictive Sciences (CAAPS), University of Augsburg, Augsburg, Germany

**Keywords:** Image analysis, Dental plaque, Co-localization, Neural network, Cellpose, Fluorescence imaging, Image processing, Machine learning, Peri-implantitis

## Abstract

Dental implant-associated infections increase the risk of implant failure, presenting significant challenges in modern dentistry. The host-microbe interaction plays a crucial role in the development of implant-associated infections. To gain a deeper understanding of the underlying mechanisms, numerous studies have been conducted using in vitro co-culture models of bacteria and human cells or in situ samples. Due to the complexity of the images generated throughout these studies, however, the analysis by means of classical image processing techniques is challenging. This study proposes a workflow—based on two custom Cellpose models—that, for the first time, allows the analysis of microbial surface coverage in microscopy images of fluorescent-stained and co-localized microorganisms and human cells with substantial background signals. The first Cellpose model demonstrated its efficacy in the analysis of individual bacteria within images derived from an 3D implant-tissue-oral biofilm in vitro co-culture model. In combination with the second custom model, which was trained to recognize microcolonies, images obtained from an in situ study could also be automatically segmented. The model’s segmentation accuracy could be further enhanced by acquiring additional training images and improving image quality, making the proposed workflow now valuable for a range of dental implant-related and other co-culture images.

**Supplementary Information:**

The online version contains supplementary material available at 10.1038/s41598-025-05484-1.

## Introduction

Microorganisms residing in the human oral cavity have the ability to adhere to surfaces (such as teeth or dental implants) and induce the formation of highly tolerant biofilms^1,2^. Mature biofilms can provoke infections in both the soft and hard tissues surrounding dental implants, potentially resulting in implant failure and necessitating implant removal^3^. To deepen our understanding of implant-associated infections and to test innovative approaches for their prevention and treatment, a variety of in vitro and in situ models have been developed. Ideally, in vitro models should contain all components involved in infection development—namely, tissue cells, oral bacteria, and the implant material. One example of such a model is the 3D implant-tissue-oral bacterial biofilm model INTER_b_ACT, where an artificial oral mucosa with integrated titanium implant is co-cultured with an oral multispecies biofilm^4^. By contrast, in situ grown biofilms are typically collected from volunteers who carry individualized splint systems^5,6^. These splints are equipped with the implant material of interest, which faces oral biofilms and mucosal tissue simultaneously, thereby enabling the adhesion of both microorganisms and human cells. For analysis of these biofilms, fluorescence staining and microscopy techniques are often utilized—for example, confocal laser scanning microscopy (CLSM)^7^. Yet the most commonly utilized fluorescent dyes unspecifically bind to DNA, and are thus not selective for bacteria^8^. This means that when bacteria and cells are kept in a co-culture, these dyes will stain both cell types identically, making it very challenging to stratify based on fluorescence intensity. To overcome the limitations of such nonspecific fluorescent dyes and enhance the accuracy of biofilm analysis, innovative image analysis techniques have become increasingly important.

The analysis of fluorescence images for cellular colonization is typically done using a digital image processing called cell segmentation. This procedure can be used to obtain information about a cellular population’s morphological characteristics or cell count from biological images. To prevent researchers from having to manually label and deal with large amounts of data, several image processing algorithms have been developed. Traditional image-processing techniques, including intensity thresholding and watershed-based algorithms, are limited in their ability to effectively distinguish individual cells in crowded images, images with large background signals, or varying fluorescence intensities^9,10^. By contrast, more recent strategies predominantly involve machine learning approaches, including the use of deep neural networks^10,11^ that are more suitable for this task.

One innovative tool for biological image segmentation is Cellpose, which was first introduced in 2021. This software utilizes a U-net-like neural network to predict topological maps, with the final segmentations achieved via gradient tracking^12^. In addition to a variety of pretrained models, users also have the option to train custom models to achieve optimal segmentation accuracy for their own specific type of images^13^. Although Cellpose was initially designed for the segmentation of eukaryotic cells, there are several examples of researchers utilizing it across diverse applications, including the detection of autophagic bodies in yeast vacuoles^14^ and the quantification of ice crystal size and shape^15^. To enhance segmentation accuracy for irregularly shaped morphologies, Omnipose was introduced in 2022. Building on the foundation of Cellpose, Omnipose incorporates distinct methods for network predictions and mask reconstructions, specifically aiming to optimize performance in bacterial segmentation^16^. However, neither of these programs has been applied to images of co-cultured human cells and microorganisms before.

The aim of this study was to develop a digital workflow that enables the quantification of the microbial surface coverage within CLSM images of co-localized human cells and microorganisms on titanium implant materials. To achieve this objective, images obtained from the INTER_b_ACT model, as well as an in situ study were utilized to train two custom Cellpose models, CP_I and CP_M, intended to segment single bacteria or chains of bacteria and microcolonies, respectively. Finally, the combined models were successfully tested on an additional set of in situ grown biofilms. The obtained results significantly extend the analysis possibilities within the scope of dental implant-related research but also far beyond. Through the refinement of the underlying models, the setup holds the potential for fully automated, multi-level image analysis.

## Methods

### In vitro samples from the INTERbACT model

The 3D implant-tissue-oral-bacterial-biofilm in vitro (INTER_b_ACT) model consists of a peri-implant mucosa model which is co-cultured with a multispecies biofilm. The peri-implant mucosa was prepared as previously reported^17^. In summary, human gingival fibroblasts (HGFs; 4 × 10^5^ cells/model, 121 0142, Provitro GmbH, Berlin, Germany) were mixed with collagen type 1 hydrogel (bovine type-I collagen, 3 mg/mL, PureCol®, 5005-100ML, Advanced Biomatrix, Carlsbad, USA), fetal bovine serum (FBS), L-glutamine (G7513, Sigma-Aldrich, St. Louis, USA), 10 × DMEM (P03-01510, Pan-Biotech), reconstitution buffer (2 mg/mL sodium bicarbonate, 2 mM HEPES, and 0.0062 N NaOH) and poured into culture inserts (3414, Corning B.V. Life Sciences, Amsterdam, Netherlands). The HGF-hydrogels were then cultivated for 4 days in a humidified atmosphere at 37 °C and 5% CO_2_. Subsequently, titanium disks (3 mm diameter, 2.7 mm height, grade 4) were inserted, and, after a three-day cultivation period, oral keratinocytes (OKF6/TERT-2; 1 × 10^6^ c/mL^18^) were then added on top of the HGF-hydrogel. The models were raised to an air-liquid interface and further cultivated in a specific airlift medium (3:1 DMEM [P04-03591, Pan-Biotech] and Ham’s F-12 [P04-14559, Pan-Biotech], 5 μg/mL insulin, 0.4 μg/mL hydrocortisone, 2 × 10^–11^ M 5 triiodo-L-thyronine, 1.8 × 10^–5^ M adenine, 5 μg/mL transferrin, 10^–10^ M cholera toxin, 2 mM L-glutamine, 10% v/v FBS, 1% v/v penicillin/streptomycin) at 37 °C in a 5% CO₂ humidified atmosphere for 15 days.

The multispecies biofilms were grown as previously described^4^. In brief, *Streptococcus* *oralis* (ATCC 9811, American Type Culture Collection, Manassas, USA), *Actinomyces naeslundii* (DSM 43,013, German Collection of Microorganisms and Cell Cultures (DSMZ), Braunschweig, Germany), *Veillonella dispar* (DSM 20,735, DSMZ), and *Porphyromonas gingivalis* (DSM 20,709, DSMZ) were cultured in brain-heart infusion (BHI) medium (Oxoid Limited, Hampshire, UK), supplemented with 10 µg/mL vitamin K, at 37 °C under anaerobic conditions. The bacteria were mixed equally in BHI with vitamin K so that each species would be present with an optical density at 600 nm of 0.01. Subsequently, the bacteria were grown under anaerobic conditions at 37 °C to form biofilms on glass cover slips (10 mm diameter, thickness 1, Fisher Scientific GmbH, Schwerte, Germany) in 24-Well plates. After 48 h of cultivation, both the mucosa model and the biofilms were washed with PBS. Thereafter, the glass cover slips were placed on the titanium of the mucosa model with the biofilm side facing the titanium implant and cultured for 48 h at 37 °C in a humidified atmosphere with 5% CO_2_.

### In situ samples from splints worn by volunteers

The study was approved by the ethics committee of the Hannover Medical School (ethical vote no 4348). The experiments were performed according to the relevant guidelines and regulations. Ten volunteers between the ages of 21 and 38 were included in this study after giving their informed and written consent and verifying their oral health by periodontal screening. The volunteers were non-smokers and did not undergo antibiotic treatment four weeks prior to the beginning of the study. For each volunteer, an individual splint was fabricated from thermoforming polyethylene (PE; Erkodur transparent, Erkodent Erich Kopp GmbH, Pfalzgrafenweiler, Germany) adapted to the lower jaw. These splints contained buccally-facing PE blocks on each side where titanium disks (3 mm diameter, 1.5 mm height, grade 4) could be individually inserted without direct contact with the cheeks or one another. Titanium samples were fixed with adhesive wax and protected from accidental swallowing by an additional thin plastic bridge. Throughout the duration of this study (8 days), the splints were worn by the volunteers and removed exclusively for the purposes of eating, drinking (with the exception of water), and teeth brushing. To avert the drying out of biofilms, the splints were placed in a 0.9% NaCl solution during that time. The splint itself was not cleaned. At 48-h intervals, one individual titanium disk was retrieved from each splint for subsequent microscopic analysis.

### Fluorescent staining and microscopic analysis

Both titanium disks from the in vitro and in situ sampling were stained using the LIVE/DEAD® BacLight™ Bacterial Viability Kit (Fisher Scientific GmbH). The fluorescent dyes Syto9 and propidium iodide (PI) were diluted in phosphate buffered saline (PBS; Biochrome GmbH, Berlin, Germany). For the samples derived from the mucosa model, a 1:2000 dilution was applied, and the samples were incubated for 30 min at room temperature. Samples from the splints were treated with a 1:1,000 dilution of fluorescent dyes and incubated for 15 min at room temperature. Subsequently, all samples were fixed for 30 min at 4 °C using 2.5% (v/v) glutardialdehyde in PBS and then covered with PBS for microscopy.

All samples were analyzed by CLSM (Leica TCS SP8, Leica Microsystems, Mannheim, Germany) at 400-fold magnification. While the excitation wavelength of the laser was set to 488 nm for Syto9, a wavelength of 552 nm was used to excite PI. To detect the emissions, the detector was set to wavelengths of 500–540 nm and 675–750 nm for Syto9 and PI, respectively. For each sample of the mucosa model, image stacks with a z-step size of 2 µm were taken at four separate positions from the top as well as from the side of the titanium disk. Image stacks with a z-step size of 2 µm were recorded at five positions for each sample deriving from the volunteer’s splints.

### Digital image preprocessing

The raw images acquired via CLSM were stored in the lif format, with each featuring a z-stack of multiple sub-images with dimensions of 1024 × 1024 pixels. Independently of the channels recorded, preliminary experiments revealed that the green Syto9 channel was the most suitable for further analysis. Maximum intensity projections were generated by transferring the highest pixel intensity values along the z-stack (x, y plane) into a single composite image. Subsequent image enhancement procedures included flatfield correction, executed with a sigma value of 50 using MATLAB’s ‘imflatfield’ function, followed by contrast enhancement through the ‘imadjust’ function (MATLAB version 24.1.0 [R2024a], The Mathworks Inc., Natick, USA).

### Training custom cellpose models using in vitro images

To develop the first Cellpose model (CP_I) capable of accurately segmenting individual bacteria or chains of bacteria that are not part of microcolonies or biofilms, a total of 46 preprocessed images from the INTER_b_ACT model were used. Of these images, 23 were captured from the top of the titanium disk and 23 from the side. The images were segmented using Cellpose 2.0, employing a human-in-the-loop approach (Cellpose version 2.3.2^13^). Prior to the labeling procedure, images underwent manual quality control. This step involved discarding any images in which distinguishing between background noise and microorganisms proved to be unfeasible. For this purpose, and given the absence of a suitable pre-existing model from the available model zoo, manual segmentation was conducted on the first image. In brief, a first Cellpose model was trained from scratch over 500 epochs, segmenting the green channel. The following settings were employed for the training of all Cellpose models presented in this study: Learning rate: 0.1, weight decay: 1 × 10^–5^, batch size: 8, solver: Stochastic Gradient Descent. No image augmentation strategies were employed, and the training was terminated upon reaching the maximum number of epochs.

This model facilitated the segmentation of the subsequent image, which was manually corrected before training a new model. This iterative process was then continued until a model capable of reliably identifying a satisfactory proportion of bacteria within the images was achieved. Individual bacteria not in contact with adjacent cells were labeled separately, whereas clusters or chains of bacteria were designated as a single region of interest (ROI).

Following the complete segmentation of the dataset, the images were divided into training and testing data subsets. Using the training images, a new Cellpose model was trained from scratch over 3,000 epochs, saving the model every 100 epochs. The accuracy (Acc; Eq. 1) of each resultant model was assessed by applying the models to segment the images in the test data set and comparing the generated segmentation masks to the ground truth masks. In this context, a true positive (TP) was defined as a pixel correctly identified as belonging to a microorganism.

1 $${\text{Acc}} = { }\frac{{{\text{True}}\;{\text{Positive}}}}{{{\text{True}}\;{\text{Positive}} + {\text{False}}\;{\text{Positive}} + {\text{False}}\;{\text{Negative}}}}$$

In addition to the custom Cellpose models, pre-trained models from the Cellpose model zoo were also evaluated for the test data set. For this purpose, the Cellpose models ‘bact_phase_cp3’, ‘bact_fluor_cp3’ and ‘deepbacs_cp3’ (Cellpose version 3.1.0^19^) were employed to segment the test data set, and the accuracy was calculated as described above. The diameter for the segmentation was set to the respective models’ default value as well as to 7.06 pixels, the mean diameter of the ROIs in the training data set.

Using the correct segmentations of the training set and the test set, the microbial surface coverage was calculated using Eq. (2) for the two image groups of the INTER_b_ACT model (images derived from the top and the side of the titanium disk). Statistical analysis was performed using Welch’s t-test.

2 $${\text{cov}} = { }\frac{{{\text{Number}}\;{\text{of}}\;{\text{pixels}}\;{\text{belonging}}\;{\text{to}}\;{\text{microorganisms}}}}{{{\text{Total}}\;{\text{number}}\;{\text{of}}\;{\text{pixels}}}} \times 100\%$$

In order to assess the effectiveness of the CP_I model in segmenting images deriving from sources other than the in vitro model presented in this study, two independent datasets were selected. The first dataset was published in the Cell Tracking Challenge (https://celltrackingchallenge.net/) and comprises a series of time-lapse images of pancreatic stem cells^20^. The CP_I model was used to automatically segment 40 images of the dataset, and the accuracy was determined with the respective reference masks, as described above. Furthermore, the CP_I model was applied to a set of 35 images selected from the Electron Microscopy Particle Segmentation (EMPS) dataset^21^. Given the heterogeneity of the dataset, which comprises a variety of electron microscopy images, a selection was made to focus exclusively on images that exhibited a reasonable degree of resemblance to microorganisms. In addition to calculating the accuracy of the models predictions, the average precision (AP) values were determined for intersection over union (IoU) thresholds of 0.5 and 0.75, as described by Stringer et al.^12^. In summary, the IoU measure provides information on how well two objects overlap, where perfectly overlapping objects yield a value of 1 and no overlap results in a value of 0. The predicted segmentation masks are matched with their respective reference masks and if the calculated IoU value for one ROI exceeds a certain threshold, it is counted as a true positive. The AP value is then calculated as shown in Eq. (1).

### Establishment of three methods for analyzing in situ images

**Fig. 1:**
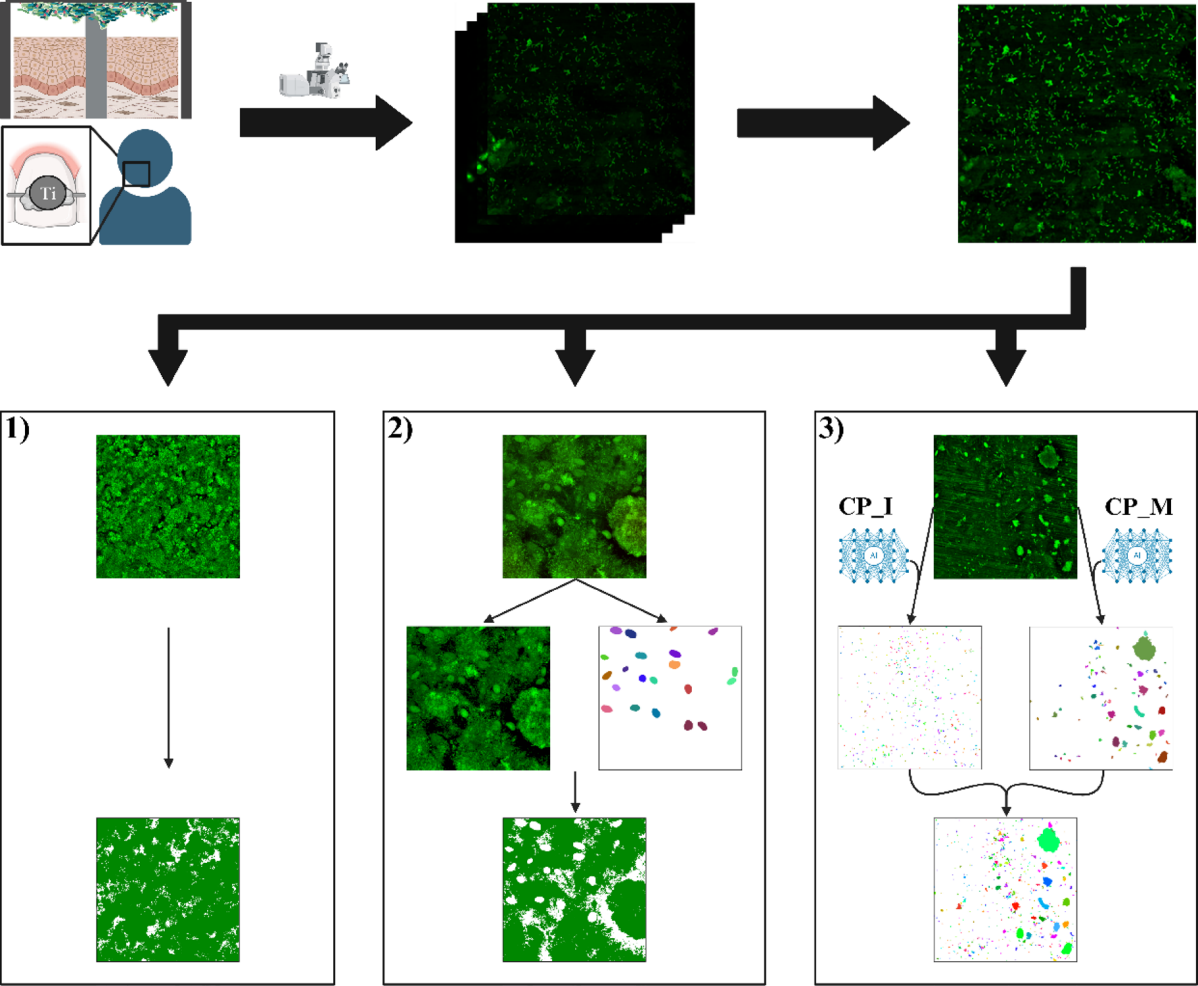
Schematic overview of the experimental workflow. CLSM images were obtained from the in vitro INTER_b_ACT model and in situ from splint-wearing volunteers. Following preprocessing, images were analyzed by three different methods, depending on image quality and content. Method 1 is a thresholding operation, which was applied for images without disturbing signals. For images with few disturbing signals, they were labeled in the Cellpose graphical user interface (GUI) and subtracted from the image before applying the thresholding operation (method 2). For method 3, two Cellpose models were used to segment the image separately. The resulting masks were combined and corrected in the Cellpose GUI (this figure was created using biorender.com).

For the preprocessed images derived from the in situ study, following the manual quality control (described above), the microbial surface coverage was quantitatively assessed using three distinct methodological approaches, which are illustrated in Fig. 1.

For images that were completely covered by biofilm, a thresholding operation was applied. Pixels exhibiting an intensity greater than 50 within the green channel were classified as microorganisms, while those with lower intensities were identified as background.

For images with minimal interference (such as background noise or human cells), an alternative method was utilized. In this approach, human cells were segmented using the pretrained model ‘cyto2’ (included in Cellpose version 2.3.2^13^) and the interfering regions were manually labeled using the GUI of Cellpose 2.0. Subsequently, the pixels within these marked regions were assigned a value of 0, after which the previously mentioned thresholding method was applied.

For images with substantial background noise and/or multiple human cells, a third method was employed. Initially, the custom Cellpose model designed to identify individual bacteria (CP_I) was utilized for image segmentation. Then, the resulting segmentation masks were manually corrected. Upon segmenting half of the dataset, a second custom Cellpose model was developed to identify microcolonies (CP_M). For this purpose, the existing segmentation masks were filtered to retain only ROIs ranging from 500 to 15,000 pixels in size. The resulting masks served as the foundation for training a new model over 1000 epochs.

For the remaining unsegmented images, both models (CP_I and CP_M) were applied separately and then combined using a Python script and manually corrected within the Cellpose GUI. These manually corrected masks of the dataset as well as the output images of the thresholding method were then used to calculate the surface coverage following Eq. (2). Statistical analysis was performed for seamless data series using a repeated measures one-way ANOVA with Bonferroni multiple comparison tests and α = 0.05.

The performance of the CP_M model was assessed by evaluating images containing microcolonies selected from the in situ dataset. The images and their corresponding corrected masks were then cropped to a size of 256 × 256 pixels, resulting in the creation of training and testing subsets containing 78 and 20 images, respectively. Based on the training set, a new CP_M model was trained for 500 epochs. Following the training process, the accuracy of the model was determined, as described above, using the testing set and Eq. (1).

In order to estimate the time saved by using the two Cellpose models as opposed to manual segmentation, a set of 12 images was selected (six images from the in vitro model and six images from the in situ study). The time required to manually label the 12 images and to correct the segmentations predicted by the two Cellpose models was measured by four test persons with varying levels of experience in image analysis. To assess the consistency of the manual corrections, the manual segmentation masks were compared to the respective reference mask for one of these images. For this, AP values were calculated for varying IoU thresholds.

## Results

### Custom cellpose model for in vitro INTER_b_ACT model image analysis

In the INTER_b_ACT model, peri-implant mucosae with integrated titanium implants were cultivated in the presence of oral multispecies bacterial biofilms. For the analysis of bacterial implant colonization, titanium disks were removed from the model after live/dead fluorescent staining and images were obtained via CLSM. These images were subsequently preprocessed to facilitate further analysis.

The in vitro dataset comprised 46 images and a total of 15,381 ROIs. Of these, 35 images were utilized as a training set for the custom CP_I model, while the remaining 11 images were reserved as a testing set. A manual evaluation of the images revealed the presence of bacteria in varying amounts with a heterogeneity of background signals in both groups. The majority of the images exhibited the coexistence of human cells and bacteria, with the latter present either as individual cells or as chains.

**Fig. 2:**
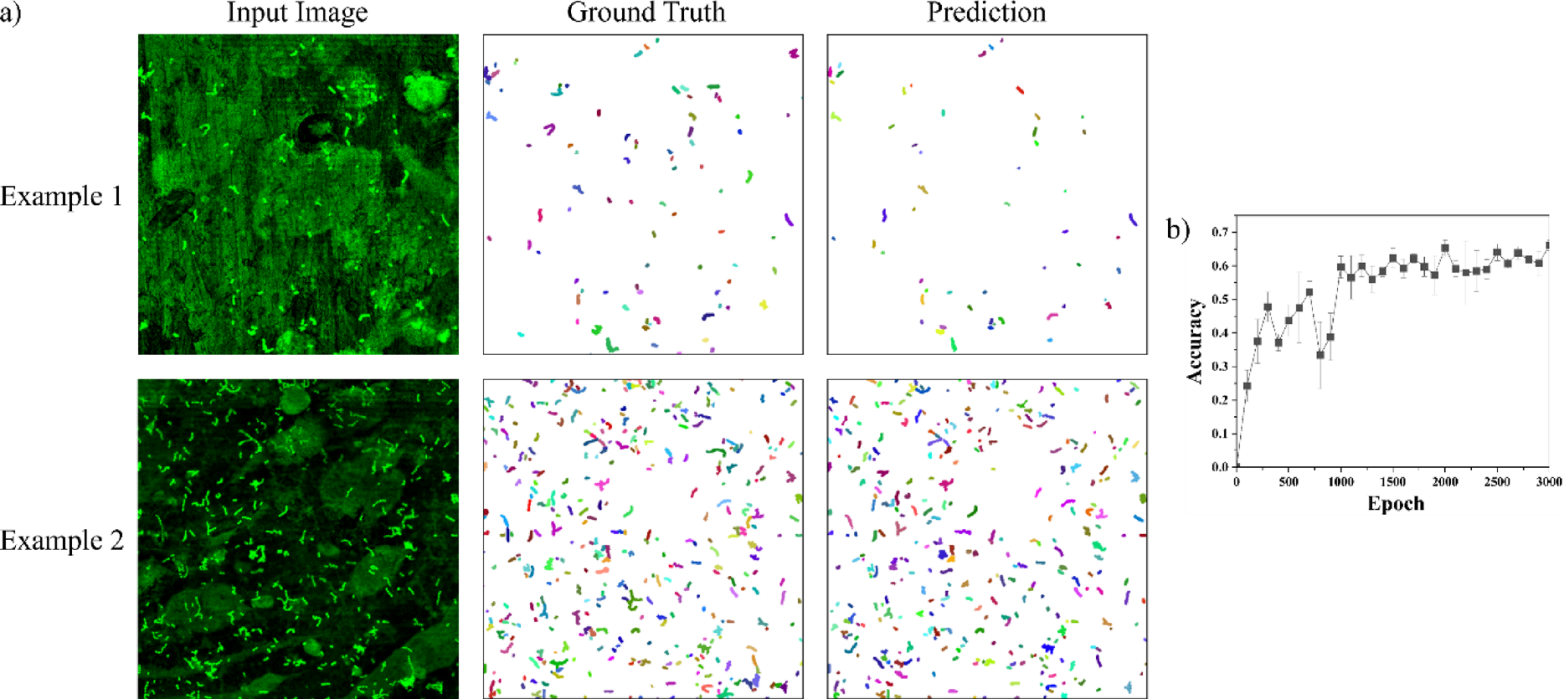
Comparative analysis of example images from the INTER_b_ACT model and segmentation outcomes. (**a**): Example images obtained from the top of the titanium implant of the INTER_b_ACT model and their corresponding ground truths and segmentation results achieved using a custom Cellpose model. (**b**): Mean ± standard deviation of the accuracy of the custom Cellpose models throughout the training process, evaluated using a test dataset that was not involved in the training process.

Figure 2A provides two examples from the test set alongside their respective correct segmentations and the predicted segmentations generated by the model with the highest accuracy. The average accuracy across five independently-trained CP_I models exhibited a significant increase within the initial 300 training epochs (Fig. 2B), ultimately reaching a plateau after 1,000 epochs. The model achieving the highest test accuracy of 0.68 was attained after 3,000 epochs of training. Of the pretrained Cellpose models evaluated, ‘bact_fluor_cp3’ demonstrated the highest performance, attaining an accuracy of 0.15.

Utilizing the segmentation masks generated by the CP_I model, a surface coverage of 3.47% (± 3.92%) and 0.05% (± 0.08%) was calculated for images captured on the top and on the lateral surfaces of the titanium disks from the INTER_b_ACT model, respectively. The difference in surface coverage was found to be statistically significant (adjusted *p*-value = 0.0005).

The CP_I model was also tested for its capacity to segment images derived from other sources. For a set of pancreatic stem cell images published in the Cell Tracking Challenge^20^, an accuracy of 0.67 (± 0.09) was achieved. This image dataset consists of time series images, with images captured at early time points exhibiting a lower cell density compared to those taken at later stages, where cells are more densely packed. For images within the first 20% of the time series, an accuracy of 0.75 (± 0.03) was obtained. For a subset of images from the EMPS dataset, which contains electron microscopy images from diverse sources, the CP_I model exhibited an accuracy of 0.77 (± 0.10) and AP values at IoU thresholds of 0.5 and 0.75 of 0.84 (± 0.16) and 0.65 (± 0.24), respectively.

### Combined threshold and custom Cellpose model analysis for in situ biofilms

**Fig. 3:**
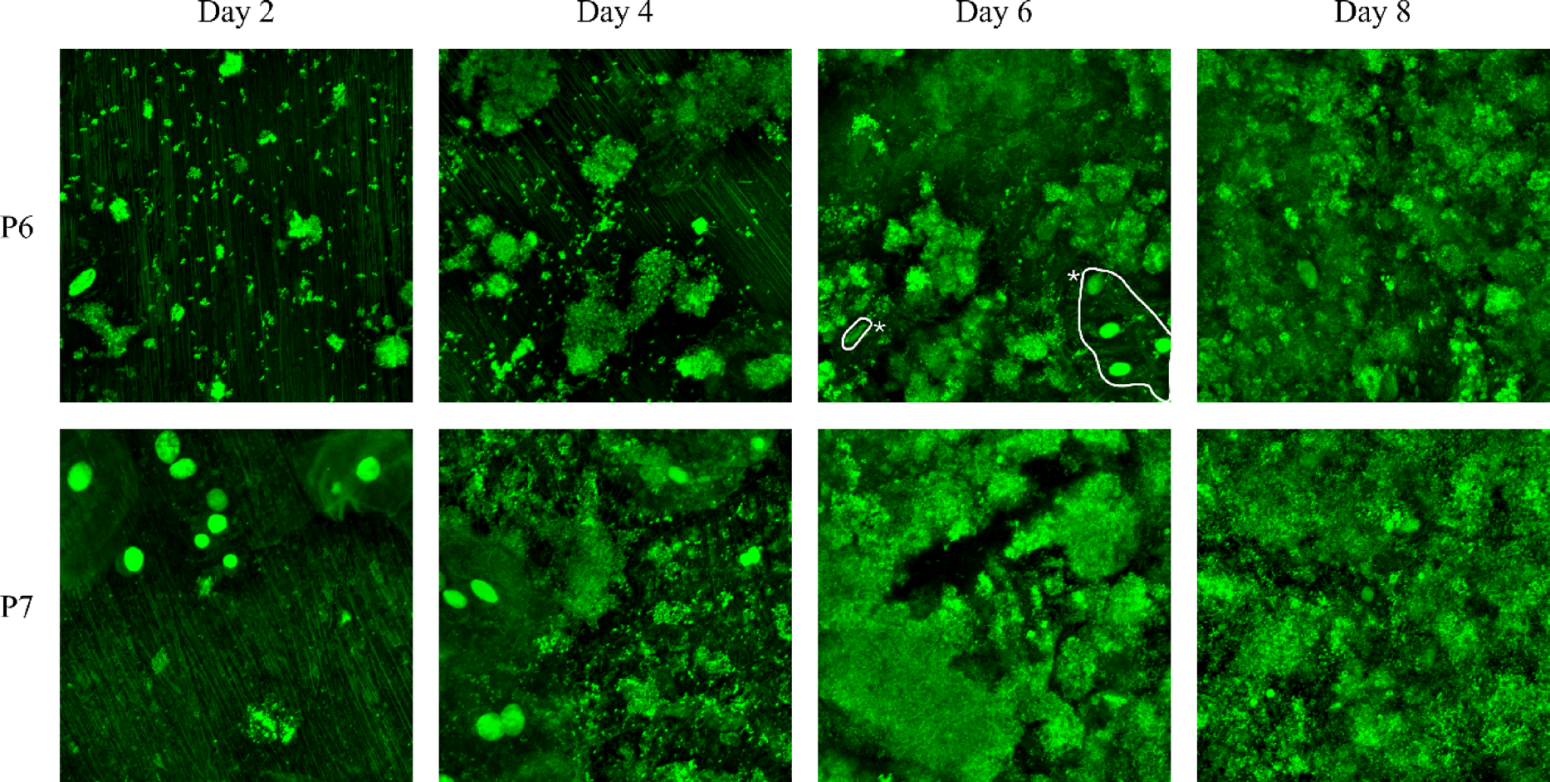
Example images (preprocessed) of in situ grown biofilms obtained by CLSM over the course of 8 days. For P6 Day 6, the areas that were subtracted during analysis are marked with an asterisk (*).

In situ grown biofilms on titanium disk-equipped splints were retrieved at two-day intervals from each participant and subjected to live/dead fluorescent staining, followed by imaging via CLSM. Across the study, a total of 176 images were preprocessed for analysis. Example images from two participants are shown in Fig. 3. The titanium disks retrieved on day 2 showed predominantly single microorganisms, smaller chains or small aggregates of microorganisms. By day 6, however, most images revealed an extensive surface coverage by biofilms.

**Fig. 4:**
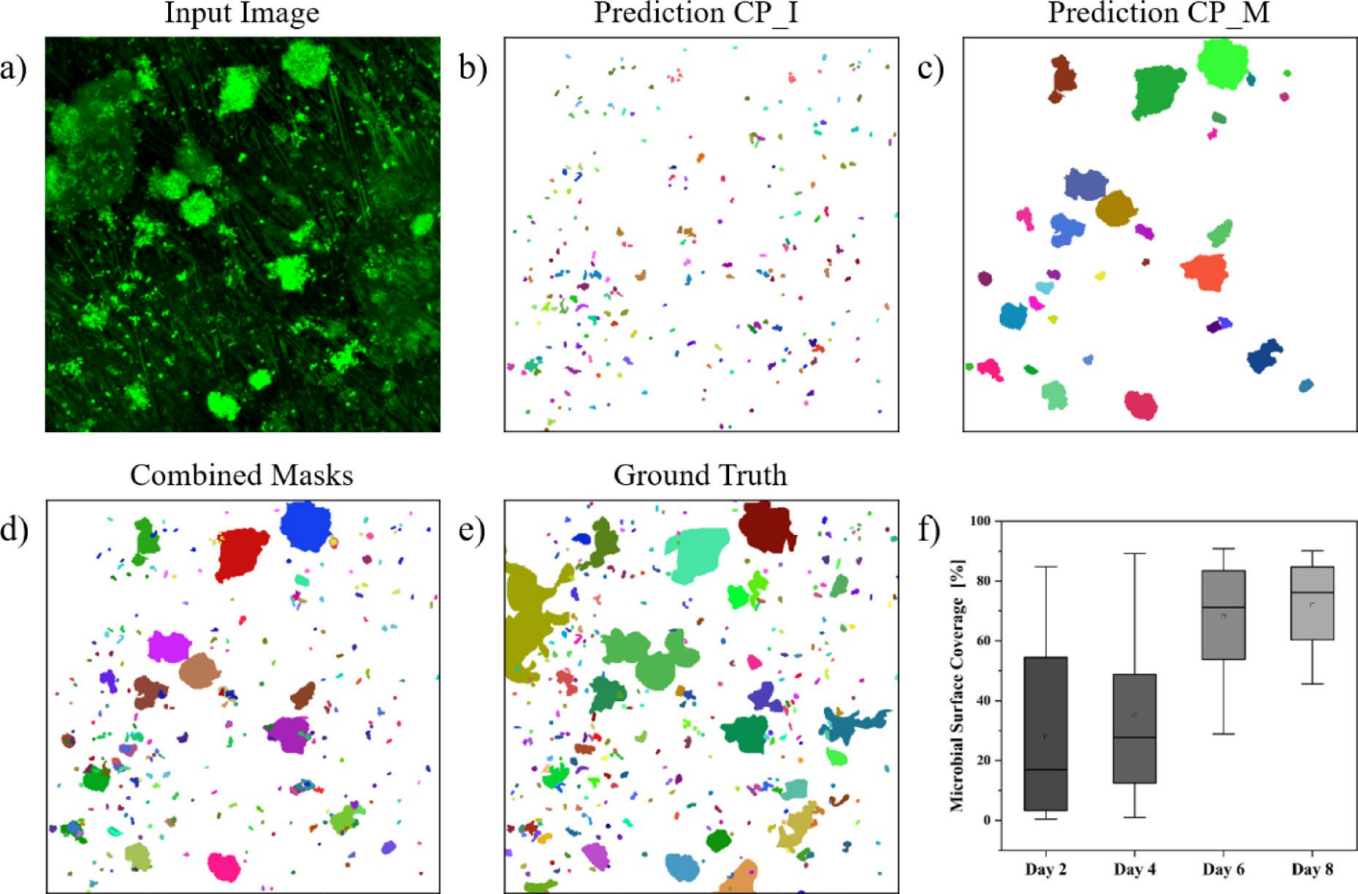
Analysis of in situ study images. (**a**): Example image from the in situ study (preprocessed and cropped to 512 × 512 pixels). (**b**): Segmentation results of the CP_I model. (**c**): Segmentation results of the CP_M model. (**d**): Combination of both predicted masks. (**e**): Ground truth of the example image. (**f**): Boxplot of the microbial surface coverage of all images over the course of 8 days.

To quantify the microbial surface coverage, three distinct methods were employed, each one tailored to the specific quality and characteristics of the image being analyzed. The first method involved a thresholding operation, which was applied to images that were entirely covered by biofilm (compare Fig. 3, P7, day 6). For images exhibiting minimal unwanted signals in addition to the microorganisms (compare Fig. 3, P6, day 6), a second strategy was adopted. Prior to applying the thresholding operation, unwanted areas were labeled in the Cellpose GUI and subtracted from the image. The third method was applied for images with substantial background noise or areas where the background could not easily be labeled (as shown in Fig. 4A). In these cases, the images underwent segmentation through two separate Cellpose models (Fig. 4B,C). The resulting masks were combined (Fig. 4D) and corrected in the Cellpose GUI to give the final segmentation (Fig. 4E). Out of the 176 images, 14 were analyzed using the thresholding method, while 79 and 83 images were evaluated using the second and third methods, respectively. The selection of the appropriate method was made manually on an image-by-image basis.

For small ROIs of less than 101 pixels, the CP_I model demonstrated an overall segmentation accuracy of 0.63 for images assessed using the third method. Notably, the accuracy for the example image (Fig. 4B) was 0.75. The accuracy of the CP_M model for ROIs of 101 or more pixels was calculated based on a testing subset generated from images of the in situ dataset and was found to be 0.74.

Based on the corrected masks (method 3) and the results of the thresholding operations (methods 1 & 2), the microbial surface coverage was calculated for all images (Fig. 4F). The mean surface coverage on day 2 was 27.81% (± 27.84%), which increased over time—eventually reaching 71.88% (± 15.25%) by sampling day 8. A substantial variation was observed among samples from different persons, however, particularly during the initial two sampling periods. While some images displayed only a few individual microorganisms, others were nearly fully covered. In general, most surfaces became densely populated with microorganisms by day 6. Statistically significant differences were identified between the surface coverages of day 2 and day 8 (adjusted *p*-value = 0.02).

A test data set comprising 12 images was utilized to demonstrate the efficacy of the trained Cellpose models. The utilization of these models resulted in a 70.13% (± 15.11%) reduction in the time required for manual corrections, as compared to the time necessary for manual labeling of the images without the assistance of the Cellpose models. In order to examine the consistency in the manual corrections between different researchers, a representative image was selected and the manual segmentations were compared to the reference mask (Supplementary Figure S1). At IoU thresholds of 0.5 and 0.75, the AP values of the manual segmentations were found to be 0.94 (± 0.01) and 0.67 (± 0.09), respectively. The maximum difference in the AP value between two researchers was 0.25 at an IoU threshold of 0.75.

## Discussion

Implant-associated infections pose a major challenge to modern dentistry. Numerous studies have been conducted to enhance our comprehension of the underlying mechanisms, which involve complex interactions between co-localized microorganisms and human cells. In this effort, extensive imaging datasets are generated, which, due to their complexity, necessitate analysis techniques that conventional image processing methods cannot readily accommodate.

In this study, we developed a workflow suitable for the semi-automated analysis of the bacterial surface coverage on titanium surfaces exposed to the human oral microbiome and oral tissue—something which would simply be infeasible to conduct manually^4–6,22,23^. For the analysis, three distinct methodologies were employed, each tailored to specific image content and quality. For images that were entirely covered with biofilm, a thresholding operation was performed. For images predominantly comprising microorganisms, i.e., with only a few areas of background or human cells present, the respective areas were marked in the Cellpose 2.0 GUI and subtracted from the image prior to applying a thresholding operation. A third method was used for images in which the interfering elements could not be separated from the microorganisms. To address this challenge, a Cellpose model was trained to recognize individual microorganisms using co-culture images from the INTER_b_ACT model (CP_I). Additionally, a separate model was trained using microscopy images from in situ biofilms to recognize microcolonies (CP_M). The resulting segmentation masks from these two models were then combined to minimize the manual labeling effort.

The images employed in this study originate from one of two sources: the INTER_b_ACT model and orally exposed test specimens. Both of these datasets featured microorganisms that co-localized with human cells on titanium surfaces, and which were subjected to live/dead fluorescent staining. However, due to the non-specificity of the fluorescent dyes (which also stain human cells and their debris), signals are generated that disturb classical image analyses. Additionally, the fluorescent dyes also tend to create background signals on the titanium surface, possibly due to unspecific binding to surface-attached proteins. And the unevenness of surfaces or cells in different z-planes can also result in variations in signal intensity across individual images. In order to prepare images for analysis, several preprocessing techniques have been described. Frequently employed methods include the reduction of noise within images through filtering, such as by applying Gaussian filters^24^. However, due to the inhomogeneity of the images in our dataset, the preprocessing for all three analytical methods included the application of flat-field correction and contrast enhancement to address the aforementioned issues. This approach obviated the need to manually set thresholds on an image-by-image basis and eliminated the necessity for repeated contrast adjustments to compensate for intensity variations.

A variety of image segmentation tools are available, which include Stardist^25^, CellProfiler^26^, DeepCell^27^, and others. Additionally, there are tools such as COMSTAT^28^ or BiofilmQ^29^ that have been developed specifically for the analysis of biofilms. While numerous of these tools might be applicable to our study, due to the inhomogeneity and substantial background noise present in our images, we elected to utilize Cellpose in this investigation. This decision was made on the basis of several factors, including the comprehensive documentation, the intuitive GUI, and the reported capacity to segment objects of varying morphologies^12^, as well as encouraging preliminary outcomes using Cellpose^23^. With images derived from the INTER_b_ACT model, a custom Cellpose model (CP_I) was trained to recognize individual microorganisms or chains of bacteria. While small ROIs have comparatively diminished impact on the surface coverage in comparison to microcolonies due to their size, their high abundance (up to several thousand per image) rendered the manual annotation process notably time-consuming. The efficacy of the CP_I model was assessed with respect to its ability to automatically segment a set of test images from the INTER_b_ACT model that were not part of the training process. The accuracy of this segmentation was found to be approximately 0.68. This suggests that overfitting did not occur despite the high number of training epochs employed—which was higher than what is typically observed in other studies^14^. The achieved accuracy was, thus, below that described in the literature for other Cellpose applications^12^. Spahn et al. investigated the performance of several available tools in regard of their ability to segment *Bacillus subtilis* cells in fluorescence images, obtaining precision values between 0.44 and 0.88^9^. For fluorescence images containing *Escherichia coli* cells, it was demonstrated that the Bacterial Cell Morphometry 3D (BCM3D) tool, presented by Zhang et al., could outperform comparable segmentation tools. Interestingly, it was shown that the segmentation accuracy is dependent on the cell density and the signal to background ratio of the images, resulting in comparable accuracies for unfavorable conditions as observed in the present study^10^. Therefore, we assume that the accuracy of the CP_I model can be attributed to the diverse shapes and sizes of the bacteria and their aggregates present in the dataset, as well as to the significant and irregular background which frequently resulted in false positive predictions. Because the automatically generated masks are subsequently manually corrected, the resulting segmentations can still be considered reliable. The microbial surface coverage calculated for the INTER_b_ACT dataset exhibited statistically significant higher values for images captured at the top of the titanium cylinders in comparison to those acquired at the cylinder’s side. This finding suggests that bacteria migrate from the top (which is directly in contact with the models’ biofilm component) along its side towards the oral mucosa component, which would directly resemble the clinical situation.

The CP_I model has also demonstrated its effectiveness in segmenting smaller microorganisms in images derived from the in situ model, achieving an accuracy of approximately 0.62. Given the minimal discrepancy in accuracy between the CP_I model and the in vitro model on which it was trained, it can be concluded that the CP_I model is also applicable to images containing individual oral microorganisms deriving from sources other than the in vitro model. For the in situ study, however, at later sampling times, the images were predominantly covered by microcolonies or larger biofilm areas which could not be detected with this model alone. To address this limitation, the segmentations of the CP_I model were combined with the masks generated by a second Cellpose model (CP_M) that was trained to recognize microcolonies. This strategy led to a substantial reduction in the manual correction effort, requiring 70% less time in comparison to the fully manual labeling of the images. The CP_M model demonstrated an accuracy of 0.74 in segmenting microcolonies within a subset of training images generated from the in situ dataset, which is slightly higher than the segmentation accuracy of individual microorganisms of the CP_I model. While the efficacy of the proposed methodology has been demonstrated for a limited number of images, we note that further refinement with a larger dataset could be beneficial to optimize the CP_M model in future. Furthermore, in order to ensure precise segmentation outcomes, manual corrections were still required.

The consistency of these manual corrections was examined by comparing the manual segmentation masks generated by different researchers with the reference mask. For IoU thresholds of 0.5 and 0.75, AP values of 0.94 and 0.67 were observed, respectively. This difference indicates that the researchers identified the same cells, but interpreted the cell boundaries slightly differently, resulting in lower AP values at higher IoU thresholds. This finding is supported by the higher standard deviation of 0.09 at an IoU threshold of 0.75, with a maximum disparity in the AP value of 0.25 between two individual researchers. This underscores that although the use of the Cellpose GUI is intuitive and straightforward for users with and without expertise, the manual effort required for correcting segmentation masks should be minimized. In addition to the considerable time savings, this would also result in more consistent segmentation outcomes, given the variability in manual corrections among individual researchers.

The CP_I model was furthermore applied to images originating from two other sources than the datasets generated within the present study. While the achieved accuracy for the segmentation of a subset of pancreatic stem cell images was observed to be comparable to the aforementioned values, it should be noted that the model performed better (acc = 0.75) for images obtained at early time stages. The cells in these images were predominantly present as individual cells and not as aggregates or touching objects. Given that the CP_I model was developed to recognize individual cells and not microcolonies, these results are consistent with the model’s functionality. Additionally, the CP_I model was employed to segment electron microscopy images from the EMPS dataset. The accuracy of 0.77, as well as the average precision of 0.84 (IoU threshold 0.5) and 0.65 (IoU threshold of 0.75), indicate that the performance of the model could be enhanced by increasing the image quality, as the EMPS images show a relatively high resolution and very low background signals in comparison to the images generated in this study. The overall segmentation outcome for the images presented in this study could therefore be supported by enhancing the image quality through specifically selecting the CLSM settings, to eventually exclude z-planes with large background signals. Additionally, future work could focus on refining the preprocessing steps through the implementation of operations such as background subtraction or filtering methods. As the images in the presented datasets are inhomogeneous, a method that categorizes them and then applies different preprocessing steps depending on the background style could be beneficial.

Due to the inhomogeneity of the images in question—particularly those derived from the in situ study depicting the known variability between persons^22^—it was necessary to select between one of the three analysis approaches presented above. While the thresholding operation is less time-consuming in comparison to the other two methods, it can only be applied to images that are free of any disturbing signals. Furthermore, additional processing steps are required to obtain morphological information about touching microorganisms. The second method is especially useful for images with few background signals and many human cells present. While the background signals required manual labeling, the human cells could be detected with high accuracy by the pretrained Cellpose models. Although both approaches are faster than the custom Cellpose model approach, they do not contribute to the objective of achieving fully automated segmentation by generating additional training data. A potential solution to this issue would be to further process the images with other image analysis tools, such as BiofilmQ. To extend the image dataset, new experiments could be performed, or existing images could be modified using image data augmentation methods^30^. Nevertheless, in general, the combination of the three methods was found to be adequate for the analysis of microbial surface coverage within in situ study-derived images. The results demonstrated an increase in surface coverage during the initial sampling times and the attainment of nearly fully covered surfaces by sampling day 6, indicating the rapid growth of early biofilms on dental implants. The values obtained in this study are consistent with published results of Azzola et al., who observed surface coverages of 28% and 65% on implant surfaces worn by a volunteer after 2 and 5 days, respectively^31^. While the surface coverage alone cannot be considered a sufficient measure for the assessment of the pathogenicity of a biofilm^32^, it can provide valuable insights into the development of biofilms, especially at early stages. Analyzing the surface coverage can, for example, be used to assess biofilm growth at different positions in the oral cavity^33^ or to identify biofilm-growth hindering properties of different implant materials or surface modifications^34^.

In conclusion, this study introduces an innovative workflow designed for the effective analysis of the cell-specific surface coverage in dental implant-related images, where traditional methods fall short, thus, addressing a critical challenge in the field of dental implant research. The study’s approach combines two custom Cellpose models, leading to the successful segmentation of microorganisms within images of co-localized microorganisms and human cells, despite the presence of significant background signals, while requiring minimal labeling effort necessary for manual corrections. Moving forward, acquiring a greater number of high-quality images, facilitated by the careful selection of microscopy settings, will be essential to improving segmentation accuracy and reducing manual labor. These advancements have the potential to contribute substantially to the broader goal of fully automated, precise segmentation, ultimately advancing our understanding of the interactions between microorganisms and human cells in dental implant applications.

## Electronic supplementary material

Below is the link to the electronic supplementary material.

Supplementary Material 1

## Data Availability

The data generated and/or analyzed in this study will be provided by the corresponding author upon reasonable request.
