# Supplementary material for: Neural network-based image analysis of co-localized microorganisms and human cells on implant materials
Source: Sci Rep. 2025 Jun 20;15:20163. doi: 10.1038/s41598-025-05484-1 (PMC12181292; doi:10.1038/s41598-025-05484-1)
Supplement: Supplementary file 1 — Supplementary Material 1 [file 41598_2025_5484_MOESM1_ESM.docx]

# Supplementary Information

# Neural network-based image analysis of co-localized microorganisms and human cells on implant materials

Nicolas Debener^1^, Anna Rosner^2,3^, Jannik Menke^1^, Carina Mikolai^2,3^, Meike Stiesch^2,3^, Katharina Doll-Nikutta^2,3#^ and Janina Bahnemann^4,5*#^

^1^Institute of Technical Chemistry, Leibniz University Hannover, Hannover, Germany

^2^Department of Prosthetic Dentistry and Biomedical Materials Science, Hannover Medical School, Hannover, Germany

^3^Lower Saxony Center for Biomedical Engineering, Implant Research and Development (NIFE), Hannover, Germany

^4^Institute of Physics, University of Augsburg, Augsburg, Germany

^5^Centre for Advanced Analytics and Predictive Sciences (CAAPS), University of Augsburg, Germany

*Correspondence: janina.bahnemann@uni-a.de

#These authors have contributed equally to this work and share last authorship


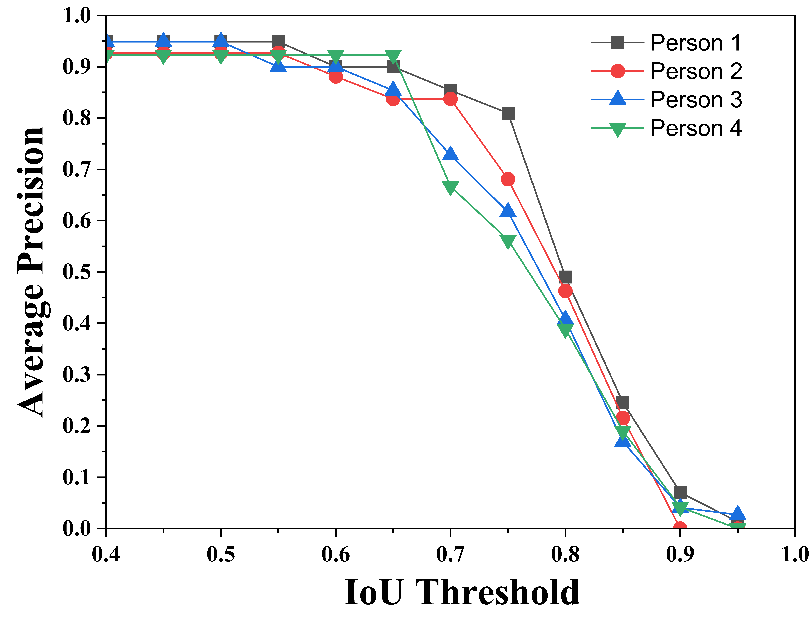


Supplementary Figure S1: Consistency of manual corrections. Average precision at different intersection over union thresholds of the separate manual segmentations conducted by four test persons for one exemplary image containing oral microorganisms.
